# Molecular biomarkers in gastro-esophageal cancer: recent developments, current trends and future directions

**DOI:** 10.1186/s12935-018-0594-z

**Published:** 2018-07-11

**Authors:** Francesca Battaglin, Madiha Naseem, Alberto Puccini, Heinz-Josef Lenz

**Affiliations:** 1 Division of Medical Oncology, Norris Comprehensive Cancer Center, Keck School of Medicine, University of Southern California, 1441 Eastlake Avenue, Suite 5410, Los Angeles, CA 90033 USA; 2 Medical Oncology Unit 1, Clinical and Experimental Oncology Department, Veneto Institute of Oncology IOV-IRCCS, 35128 Padua, Italy; 3 Oncologia Medica 1, Ospedale Policlinico San Martino, 16132 Genoa, Italy

**Keywords:** Gastro-esophageal cancer, Molecular biomarkers, HER-2, The Cancer Genome Atlas (TCGA), Asian Cancer Research Group (ACRG), Genomic profiling, Epigenomics, Immunotherapy, Liquid biopsy

## Abstract

Gastro-esophageal adenocarcinomas (GEA) represent a severe global health burden and despite improvements in the multimodality treatment of these malignancies the prognosis of patients remains poor. HER2 overexpression/amplification has been the first predictive biomarker approved in clinical practice to guide patient selection for targeted treatment with trastuzumab in advanced gastric and gastro-esophageal junction cancers. More recently, immunotherapy has been approved for the treatment of GEA and PD-L1 expression is now a biomarker required for the administration of pembrolizumab in these diseases. Significant progress has been made in recent years in dissecting the genomic makeup of GEA in order to identify distinct molecular subtypes linked to distinct patterns of molecular alterations. GEA have been found to be highly heterogeneous malignances, representing a challenge for biomarkers discovery and targeted treatment development. The current review focuses on an overview of established and novel promising biomarkers in GEA, covering recent molecular classifications from TCGA and ACRG. Main elements of molecular heterogeneity are discussed, as well as emerging mechanisms of primary and secondary resistance to HER2 targeted treatment and recent biomarker-driven trials. Future perspectives on the role of epigenetics, miRNA/lncRNA and liquid biopsy, and patient-derived xenograft models as a new platform for molecular-targeted drug discovery in GEA are presented. Our knowledge on the genomic landscape of GEA continues to evolve, uncovering the high heterogeneity and deep complexity of these tumors. The availability of new technologies and the identification of promising novel biomarker will be critical to optimize targeted treatment development in a setting where therapeutic options are currently lacking. Nevertheless, clinical validation of novel biomarkers and treatment strategies still represents an issue.

## Background

Gastric and esophageal adenocarcinomas, collectively referred to as gastro-esophageal adenocarcinomas (GEA), represents a severe global health issue. Gastric cancer (GC), in fact, ranks fifth among the most common malignancy in the world, and is the third leading cause of cancer-related death in both sexes worldwide [1]. Esophageal cancer (EC) on the other hand has a lower incident, ranking eighth among the most common cancers, but the overall mortality is fairly high (ratio of mortality to incidence of 0.88) [1]. Both malignancies are more frequent in males than in females and their incidence and mortality rates vary according to geographical regions, under the influence of several factors such as ethnicity, diet and infectious agents (i.e. *Helicobacter pylori*, Epstein-Barr Virus for GC); with the highest rates occurring in Eastern Asia. Although incidence of distal GC is declining over the past decades, incidence of upper third GC, junctional (GEJ) and lower third of the esophagus adenocarcinoma (EAC) are relatively increasing [2]. Despite recent improvements in the multidisciplinary and multimodality treatment, in fact, the overall prognosis for patients with GEA remains poor, with a global 5-year survival rate lower than 30% for GC and about 19% for EAC [3].

Moving from histo-pathological classifications, a great effort has been spent in recent years to define a genomic characterization of GEA, and to identify prognostic and predictive molecular biomarkers in order to better understand and represent the wide heterogeneity of these malignancies, and guide the development of effective targeted therapies. Major steps forward have been made for GC, with the identification first of HER2 overexpression and *HER2/neu* (*ERBB2*) amplification as predictive biomarkers for trastuzumab (Herceptin^®^; Genentech, San Francisco, California) efficacy in the metastatic setting, and more recently with the introduction of two novel genomic classifications by The Cancer Genome Atlas (TCGA) Research Network [4] and the Asian Cancer Research Group (ACRG) [5]. Thanks to this progress, different molecular subtypes of GC underlying different pathogenesis, genetic mechanisms and potentially druggable targets have been identified and novel therapeutic strategies are under development. Recently, based on microsatellite instability (MSI) and PD-L1 status as biomarkers, immunotherapy has been now integrated in the treatment of GEA. Nevertheless, the need to validate and implement promising molecular biomarkers in clinical practice is still critical in order to improve treatment selection and patients outcomes.

The present review focus on summarizing the recent developments, current trends and future perspectives on molecular biomarkers in gastro-esophageal cancer.

## Molecular biomarkers in gastro-esophageal cancer: where are we coming from

### Gastric cancer

Before the era of molecular biomarkers, GC has been classified by Lauren according to histological criteria identifying two different entities: the intestinal type and the diffuse type, plus a less common indeterminate type, with different phenotypes underlying different pathogenesis and prognosis [6, 7]. Although highly heterogeneous in treatment response, no predictive biomarker was available to guide therapeutic decisions for GCs before the discovery of HER2 overexpression/*ERBB2* amplification and the introduction of targeted anti-HER2 treatment with trastuzumab.

HER2 is a receptor tyrosine kinase (RTK) belonging to the family of epidermal growth factor receptor (EGFR) coded by the proto-oncogene *ERBB2*, which plays an important role in cell differentiation, survival and proliferation [8]. The amplification of *ERBB2* leads to an overexpression of HER2 promoting cancer cells survival, growth, migration and proliferation through the activation of the RAS/RAF/mitogen-activated protein kinase (MAPK) and the phosphatidylinositol-3 kinase/protein kinase-B/mammalian target of rapamycin (PI3K/AKT/mTOR) signalling pathways. The incidence of HER2 overexpression in GC ranges from 9 to 38% in most studies, depending on tumor location and histology [9–13], with higher frequencies in GEJ tumors and in intestinal type tumors [14–17]. The correlation between HER2 overexpression and tumor clinico-pathological features, however, is still debated, as some evidence suggests an association with cancer stage, tumor size, local invasion and nodal metastasis, not confirmed by other available data. The possible prognostic role of HER2 in GC is controversial as well. Some studies, in fact, have shown an association between HER2 overexpression and a worse prognosis, while others did not confirm a significant difference between HER2-positive and negative tumors [9, 11, 18–20]. In 2010, the international phase III randomized trastuzumab for gastric cancer (ToGA) trial, for the first time showed a significant overall survival (OS) improvement from the administration of trastuzumab, an anti-HER2 monoclonal antibody, in combination with platinum-based chemotherapy compared to chemotherapy alone in patients with HER2-positive advanced GEA (combining GC and GEJ tumors) [21]. In a post hoc exploratory analysis patients with the highest level of HER2 expression, measured as immunohistochemistry (IHC) 2+ and fluorescent in situ hybridization (FISH)+ or IHC 3+, derived the greatest benefit from trastuzumab compared to patients with low levels of HER2 expression via IHC despite a positive FISH for *ERBB2* amplification (IHC 0 or 1 and FISH positive). Based on the results of this trial trastuzumab in combination with platinum-based chemotherapy has been approved for the first-line treatment of GEA with HER2 overexpression or *ERBB2* amplification, and testing for HER2 status is recommended before starting treatment in all patients with advanced GEA who are candidates for HER2-targeted therapy [22]. Of note, recently the College of American Pathologists, American Society for Clinical Pathology, and American Society of Clinical Oncology have released official guidelines with recommendations for optimal HER2 testing and clinical decision-making in patients with GEA [23]. Results from ongoing trials investigating the activity of trastuzumab as well as a double-blockade strategy with trastuzumab plus pertuzumab, in combination with chemotherapy, in the neoadjuvant/perioperative setting (i.e. locally advanced gastric or GEJ HER2-positive tumors: NCT01196390, NCT02205047, NCT02581462), will potentially lead to further testing and treatment indications.

The anti-vascular endothelial growth factor receptor 2 (VEGFR-2) ramucirumab (Cyramza^®^, Eli Lilly and Company) is the second targeted agent which has been approved for the treatment of GC and GEJ tumors [24, 25]. To date, similar to other cancer types, no predictive biomarkers are available for anti-VEGFR treatment in GEA [26]. Of note, however, plasma levels of VEGF-A and Angiopoietin-2 (Ang-2), two well-known key drivers of tumor angiogenesis, alongside tumor neuropilin-1 expression, have been respectively reported as promising predictive and prognostic biomarkers in patients treated with bevacizumab in the phase III AVAGAST trial, investigating the addition of bevacizumab to chemotherapy in advanced GC. Interestingly, preplanned subgroup analyses showed a regional variability in these findings, possibly reflecting an underlying heterogeneity which may account, at least partially, for outcome differences observed in this trial between Asian and non-Asian patients [27–29]. Indeed, GC has the highest incidence in Eastern Asian countries (i.e. China, Japan, and Korea), however, Asian countries have consistently reported superior GC outcomes. The underlying reasons remain mostly unclear, possibly involving a complex interaction of ethnicity, epidemiological and biological factors, molecular heterogeneity and healthcare environment variability.

On the other hand, promising predictive molecular biomarkers for targeted treatments, such as *EGFR* and mesenchymal-epithelial transition factor receptor (*MET*) amplification, failed to prove their role in GC. *EGFR* is found to be amplificated in about 33% of GC, 30–60% of GEJ adenocarcinomas and 8–31% of distal EAC [30, 31], and has been evaluated as a potential target for treatment in several trials. Despite a strong rationale, anti-EGFRs, either monoclonal antibodies cetuximab and panitumumab, or small TKIs such as gefitinib and erlotinib, did not show any benefit in GEA [32, 33]. Of note, however, enrollment in these trials were not selected according to EGFR expression, thus results of an ongoing phase III trial investigating the anti-EGFR nimotuzumab as second-line treatment in EGFR IHC 2+ or 3+ recurrent or metastatic GC are awaited (NCT03400592). The prognostic impact of EGFR amplification remains controversial, as some authors have suggested a negative prognostic value [34], which has not been confirmed in other series. MET, the receptor of hepatocellular growth factor (HGF), plays a key role in several physiologic processes involving cell proliferation, survival and differentiation through the activation of multiple pathways including PI3K–AKT and RAS–MAPK signaling cascades [35]. Mutations or aberrant MET activation are associated with the development of several cancer types including GC. MET protein over-expression is present in up to 50% of advanced GC, and *MET* amplification can be found in up to 20% of GC [36], characterizing a more aggressive disease with poor prognosis [37]. Despite encouraging results in small phase II trials, MET-targeted inhibition was tested in phase III trials in MET-positive GEA with negative results [38, 39].

More recently, modern high through-put molecular technologies such as next generation sequencing (NGS) exploiting whole genome sequencing and providing more comprehensive and accurate tools for genome analysis, have become available. The use of these techniques has allowed the identification of several candidate genes mutations in known cancer-related genes in GC, such as *TP53*, *PTEN*, *ARID1A*, *APC*, *CTNNB1*, *CDH1*, *PI3KCA* and *KMT2C* [40, 41]. Moving from these data, a large effort was dedicated to defining distinctive molecular subtypes, based on genomic profiling, in order to dissect the complex heterogeneity of this disease and aid the development of novel biomarkers and targeted treatment to improve patients outcome. The TCGA and ACRG classifications, developed to address this issue, will be discussed in detail in the next sections alongside subtypes-related novel biomarkers and targeted therapies.

### Esophageal cancer

No molecular biomarker is currently approved in clinical practice for EAC excepting HER2 in GEJ cancers. Recently, however, multiple studies have explored the genomic profiling of EAC highlighting the presence of mutations in several cancer-related genes and distinctive gene signatures with could potentially translate in the development of novel biomarkers for targeted treatment. A study from Dulak et al. analyzed the genomic profile of 149 EACs using whole-exome sequencing. Main genes identified as mutated in this tumor series were *TP53* (72%), *ELMO1* (25%), *DOCK2* (12%), *CDKN2A* (12%), *ARID1A* (9%), *SMAD4* (8%) and *PIK3CA* (6%). Additionally, amplifications of several oncogenes such as *KRAS* (21%), *HER2* (19%), *EGFR* (16%), *CND1* (10%) and *MET* (6%) were identified, as well as loss of *SMAD4* (34%), *CDKN2A* (32%) and *ARID1A* (10%) [42]. Another study compared the gene signature of esophageal squamous cell carcinoma and EAC, highlighting a higher prevalence of *HER2* and *EGFR* amplification, TGF-β signaling activation and RAS/MEK/MAPK pathway activation in EAC [43]. On the other hand, PI3K/AKT/MTOR signaling, fibroblast growth factor (FGF) signaling, epigenetic regulation pathways and the NOTCH signaling pathway showed a lower frequency in EAC. Additionally, *TP53* and *CDKN2A* were highly altered in both tumor types. Finally, based on data from the International Cancer Genome Consortium project, Secrier et al. proposed a classification with potential therapeutic relevance based on a whole-genome sequence analysis of 129 EAC samples [44]. Results of the analysis showed a wide tumor heterogeneity with high prevalence of copy number alterations and frequent large-scale rearrangements. Based on their mutational signature the authors were able to identify three distinct molecular subtypes: a dominant T>G mutational pattern associated with a high mutational load and neoantigen burden (mutagenic, 53%), a C>A/T dominant mutational pattern with evidence of an aging imprint (29%) and a DNA damage repair (DDR) impaired pattern characterized by a BRCA-like enriched signature with prevalent defects in the homologous recombination pathway (18%). Co-amplification of RTKs and/or downstream mitogenic pathways was common (i.e. a simultaneous amplification of *ERBB2* and *MET*), underlining a rationale for dual targeted inhibition for the treatment of these tumors which proved to be effective in in vitro experiments by the same authors. Additionally, in in vitro models, the DDR-impaired subgroup appeared to be sensitive to DNA damage repair-targeted treatment, such as the combination of PARP inhibitors with DNA-damaging agents. WEE1/CHK1 and G2/M-phase checkpoint regulators, were also identified as potential targets in this study.

The emerging scenario for EAC is thus characterized by genomic instability with complex rearrangements leading to a significant degree of heterogeneity between patients. Although promising, however, data on genomic profiling and potential genetic biomarkers in EAC still need further validation.

**Fig. 1:**
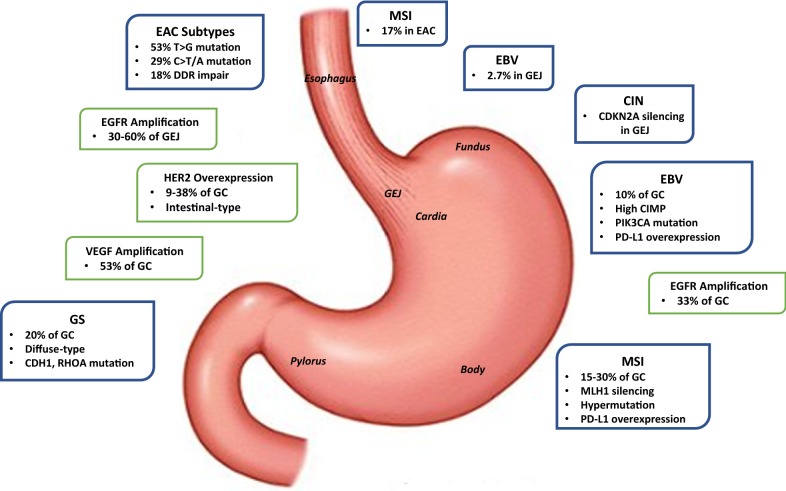
Schematic representation of main biomarkers and molecular characteristics according to tumor location and genomic subtype

Main biomarkers and trials of targeted therapies in GEA are summarized in Table 1. A schematic representation of main biomarkers and molecular characteristics according to tumor location and genomic subtype (further discussed in the next sections) are illustrated in Fig. 1.

**Table 1:** Main biomarkers and trials of targeted therapies in gastric and esophageal adenocarcinoma

Target	Biomarker	Targeted agent	Study (treatment line)	Regimen	Primary endpoint	Positive study Y/N	Refs.
HER2	*HER2* amplification/ overexpression						
		Trastuzumab	ToGA (1st)	Trastuzumab + CX vs CX	OS	Y	[21]
		Lapatinib	TRIO-013/LOGiC (1st)	Lapatinib + XELOX vs XELOX	OS	N	[95]
		Lapatinib	TyTAN (2nd)	Lapatinib + paclitaxel vs paclitaxel	OS	N	[95]
		T-DM1	GATSBY (2nd)	T-DM1 vs taxane	OS	N	[97]
		Pertuzumab	JACOB (1st)	Pertuzumab + trastuzumab + CF vs trastuzumab + CF	OS	N	[98]
VEGF-A and VEGFR-2	–						
		Bevacizumab	AVAGAST (1st)	Bevacizumab + CX vs CX	OS	N	[29]
		Ramucirumab	REGARD (2nd)	Ramucirumab vs placebo	OS	Y	[24]
		Ramucirumab	RAINBOW (2nd)	Paclitaxel + ramucirumab vs Paclitaxel	OS	Y	[25]
EGFR	*EGFR* amplification						
		Cetuximab	EXPAND (1st)	Cetuximab + CX vs CX	PFS	N	[32]
		Panitumumab	REAL-3 (1st)	Paniumumab + EOX vs EOX	OS	N	[33]
MET and HGF	*MET* amplification						
		Rilotumumab	RILOMET-1 (1st)	Rilotumumab + ECX vs ECX	OS	N	[38]
		Onartuzumab	METGastric (1st)	Onartuzumab + FOLFOX vs FOLFOX	OS	N	[39]
FGFR2	FGFR2 polysomy/gene amplification						
		AZD4547	SHINE (2nd)	AZD4547 vs paclitaxel	PFS	N	[73]
PD-1	–						
		Nivolumab	ATTRACTION-2 (ONO-4538-12) (≥ 3rd)	Nivolumab vs placebo	OS	Y	[81]
	PD-L1 expression						
		Pembrolizumab	KEYNOTE-059 (≥ 3rd cohort 1; 1st cohort 2 and 3)	Pembrolizumab (cohort 1); Pembrolizumab + CF (cohort 2); Pembrolizumab (cohort 3)	ORR (cohort 1 and 3)	Y	[80]
		Pembrolizumab	KEYNOTE-028 (after failure on standard therapy or if standard therapy not appropriate)	Pembrolizumab	ORR	Y	[82]
PD-L1	–						
		Avelumab	JAVELIN Gastric 300 (3rd)	Avelumab + BSC vs CT	OS	N	[87]

* BSC* best supportive care, *CF* cisplatin + fluoropyrimidine, *CT* chemotherapy, *CX* cisplatin + capecitabine, *EGFR* epidermal growth factor receptor, *EOX* epirubicin + oxaliplatin + capecitabine, *FGFR* fibroblast growth factor receptor, *FOLFOX* 5-fluorouracil + leucovorin + oxaliplatin, *HGF* hepatocyte growth factor, *ORR* overall response rate, *OS* overall survival, *PD-1* programmed cell death protein 1, *PD-L1* programmed death-ligand 1, *PFS* progression-free survival, *VEGF* vascular endothelial growth factor, *VEGFR* vascular endothelial growth factor receptor, *XELOX* capecitabine + oxaliplatin

## TCGA gastric cancer subtypes

In 2014, the TCGA network used six genomic and molecular platforms to comprehensively characterize 295 gastric tumors into four molecular subtypes: Epstein-Barr virus (EBV)-positive tumors (9%), microsatellite instable (MSI) tumors (22%), genomically stable (GS) tumors (20%), and tumors with chromosomal instability (CIN) (50%) [4]. Their goal was to develop a robust molecular classification of GC and to identify aberrant pathways and candidate drivers of unique classes of GC. Later, Sohn et al. [45]. conducted a follow-up study to investigate the clinical significance of TCGA subtypes. They discovered that the EBV subtype was associated with the best prognosis, and GS subtype was associated with the worst prognosis. Furthermore, patients with MSI and CIN subtypes had poorer overall survival than those with EBV subtype, but better overall survival than those with GS subtype. Sohn et al. also evaluated differences in response to chemotherapy between the four TCGA subtypes, and discovered that patients with the CIN subtype derived the greatest benefit from adjuvant chemotherapy, whereas those with the GS subtype derived the least benefit from adjuvant chemotherapy. Hence, the TCGA subtypes offer useful biomarkers for development of targeted therapies for GC patients with different prognostic outcomes and responses to chemotherapy. The four TCGA subtypes are described in detail below.

### EBV-positive

Epstein-Barr virus is a DNA virus infecting over 90% of the global population, and is currently categorized as a group-1 carcinogen associated with many cancers, including nasopharyngeal carcinomas, Burkitt’s lymphoma and Hodgkin’s lymphomas [46]. EBV was first discovered in GC in 1990, with an annual incidence of 75,000–90,000 cases per year [46]. EBV is not yet reported in esophageal adenocarcinomas; however, its prevalence in GC is approximately 10%, and in gastroesophageal junction cancers is reported to be 2.7% [47]. EBV-positive GC is more prevalent among males and younger patients [46]. Although several reports have concluded that EBV is predominantly found in proximal gastric regions [48], the TCGA cohort found EBV-positive GC to be localized to the gastric fundus or body [4]. Based on the TCGA data, EBV associated GCs have the best prognosis [45].

It is postulated that EBV enters gastric epithelia through the oropharynx and establishes a lifelong virus carrier state, called latent infection, where it persists as an episome within the nucleus and is propagated to daughter cells during cell division [46]. During latency, EBV induces extensive CpG island methylation, including both promoter and non-promoter islands of the human genome [49]. Unsupervised clustering of CpG methylation performed by TCGA revealed that all EBV-positive tumors exhibited extreme CpG island methylator phenotype (CIMP), which was distinct from that in the MSI subtype [4]. For instance, EBV-positive tumors have hypermethylation of the *CDKN2A* (p16) promoter, but lack *MLH1* hypermethylation [4]. A comprehensive analysis of promoter methylation status of 51 gastric carcinoma cases was conducted by Shinozaki and colleagues [50], who subsequently classified GCs into three epigenotypes characterized by different sets of methylation genes: EBV-positive/extensively high-methylation, EBV-negative/high-methylation and EBV-negative/low-methylation. Methylated genes specific for the EBV-positive subtype included *CXXC4*, *TIMP2* and *PLXND1*. *COL9A2*, *EYA1* and *ZNF365* were highly methylated in EBV-positive and EBV-negative/high-methylation subtypes, whereas *AMPH*, *SORC33* and *AJAP1* were frequently methylated in all epigenotypes. They discovered that EBV-positive GCs had approximately 270 genes which were uniquely methylated. Interestingly, *MLH1* was frequently methylated (46%) in the EBV-negative/high-methylation phenotype, whereas none of the EBV associated GC cases showed *MLH1* methylation. Similar results were observed in the TCGA analysis, where EBV-positive GCs lacked the *MLH1* hypermethylation characteristic of MSI-associated CIMP.

In addition to hypermethylation, EBV-positive GCs also exhibit elevated levels of programmed death ligands 1 and 2 (PD-L1/2) [46]. PD-L1 is encoded by *CD274* and PD-L2 is encoded by *PDCD1LG2*, both of which are immunosuppressant proteins inhibiting cytokine production and cytolytic activities of CD4 and CD8 T cells [51]. Therefore, inhibitors of PD-L1/2 are currently being evaluated as targets for augmenting immune response against cancer cells. Both these genes are located on chromosome 9p24.1, and were notably amplified in the EBV subtype of TCGA cohort [4]. Evaluation of mRNA by TCGA also showed increased expression of PD-L1 and PD-L2 in this subtype. This overexpression characterizes the immune signature of EBV-positive GCs, which is known to have a prominent lymphoid infiltration of the stroma and high density of tumor infiltrating lymphocytes (TILs), creating a balance between host immune evasion through PD-L1/2 overexpression, and host immune response [46]. Hence, EBV subtype is a promising candidate for anti-PD-1/PD-L1 therapy in gastroesophageal cancers.

Somatic mutations unique to EBV-positive GCs include activation of BMP (bone morphogenetic protein) signaling [52], amplification of *JAK2*, *MET*, *ERBB2*, non-silent *PIK3CA* mutations, and mutations in *ARID1A* and *BCO* [4]. TP53 mutations were rare in EBV subtype. Hence, EBV-positive GCs could be treated with drugs targeting the BMP/SMAD, JAK2, PIK3CA, MET and ERBB pathways. In the TCGA analysis, *PIK3CA* mutations were more dispersed in EBV-positive cancers, but localized in the kinase domain (exon 20) in EBV-negative cancers. TCGA investigators also reported that the two most marked features of EBV-positive cancers are diminished hypoxia-inducible factor 1α-related activity and diminished ERBB receptor signaling [52]. Furthermore, the EBV-miRNA cluster is postulated to promote cancer cell resistance to hypoxia and poor nutrient supply along with invasiveness [53]. Hence, angiogenesis inhibitors might also prove to be useful in this subtype.

### Microsatellite instability

Approximately 15–30% of GC [54], and 17% of GEJ cancer patients [55] have MSI. The MSI phenotype results from mutations in repetitive sequences due to a defective DNA mismatch repair (MMR) system [54]. This can occur in the context of hereditary syndromes, such as Lynch syndrome, with germline mutations in *MLH1*, *MSH2*, *MSH6* or *PMS2*, or it can occur sporadically through somatic mutations in MMR genes [54]. Epigenetic silencing of *MLH1* by promoter hypermethylation is the main mechanism leading to MMR deficiency in both sporadic and familial MSI GC cases.

In the TCGA cohort, most MSI patients were female (56%), and were of advanced age (median age 72) [4]. Furthermore, patients with MSI had poorer overall survival than the EBV subtype, but better than the GS subtype [45]. A German study conducted by Mathiak et al. examined 452 GC patients, and discovered that MSI was significantly more prevalent in elderly patients, distal stomach, and was associated with a significantly lower number of lymph node metastases with a significantly better overall and tumor-specific survival [56].

Similar to the EBV subtype, MSI also displays overexpression of PD-L1 [57]. Strong immunogenicity associated with MSI GC has shown improved treatment responses to PD-1 inhibitors among this subtype [58]. Hence, PD-1 inhibitors, such as pembrolizumab, are now approved for use in metastatic MSI GC s and novel immunotherapy options continue to be investigated in MSI. However, treatment benefits and prognosis may be stage dependent. For instance, the CLASSIC trial investigated 592 GC patients, and discovered that MSI status correlated with favorable prognosis in patients with stage II and III GC, but did not show benefits from adjuvant chemotherapy [59].

The successful response to immunotherapy in MSI patients may be related to increased tumor mutational burden associated with this subtype [57]. MSI is characterized by elevated mutation rates, including mutations of genes encoding targetable oncogenic signaling proteins [60]. MSI GCs have been shown to harbor more mutations in genes that act as tumor suppressors or oncogenes [61]. TCGA HotNet analysis of genes mutated within MSI tumors revealed common alterations in major histocompatibility complex class I genes, including *beta*-*2 microglobulin* (*B2M*) and *HLA*-*B* [4]. *B2* *M* mutations result in loss of expression of HLA class 1 complexes, which benefits hypermutated tumors by reducing antigen presentation to the immune system [62]. Targetable amplifications were not identified in MSI, however, mutations in *PIK3CA*, *ERBB3*, *ERBB2*, *ARID1A*, and *EGFR* were noted [4]. Integrated exome sequencing by Liu and colleagues [63] revealed that MSI GCs have frequent mutations in *TP53*, *ACVR2A*, *PTEN*, *PIK3CA*, *KRAS*, *ERBB2*, *ZBTB1*, *TRAPPC2L*, *GPR39*, *GPR85*, and *CHRM3*. Interestingly, *BRAF* V600E mutations were not observed in MSI GCs, which is commonly seen in MSI colorectal cancer [4].

### Genomically stable

When the TCGA classified tumors based on the number of somatic copy-number alterations, one of the classifications was genomically stable (GS) subtype. The GS subtype is characterized by low mutation rates and low copy-number alterations [4]. It is diagnosed at a younger age (median age 59), and has an enrichment of the diffuse histological subtype of GC [4]. As diffuse-type GC are known to be aggressive and invasive, their rapid tumor progression may result in a diagnosis at an early age and may not provide enough time to accumulate mutations [64]. Prognostically, GS subtype is associated with the worst overall survival and recurrence-free survival among the four TCGA subtypes. It has also been shown to be resistant to adjuvant chemotherapy [45].

The clinical outcomes observed in GS could be a result of the molecular landscape of this subtype. For instance, NUPR1 is an activated transcription regulator in GS subtype, and recent studies have demonstrated that it enhances chemo-resistance in multiple cancers [45]. From the TCGA data, *CDH1* (*Cadherin 1*, encoding E-cadherin) was found to be mutated in 11% of all GCs, with 37% of all GS GC having a *CDH1* mutation [4]. Genomically stable subtype also had frequent mutations in *ARID1A*, *CLDN18*, *CDH1*, and *RHOA* (*Ras homolog family member A*). *ARID1A* is a tumor suppressor encoding a subunit for switch-sucrose nonfermentable (Swi-SNF) box, and is crucial for chromatin remodeling [65]. Loss of expression of *ARID1A* has widespread implications in tumor development, and is associated with lymphatic invasion, MSI, and poor prognosis [64]. Therefore, *ARID1A* could be useful for targeted treatment potentials. *RHOA* also plays significant roles in cell migration, adhesion, cell survival, cell division, gene expression and vesicle trafficking, thereby guiding tumor cell biology [66]. However, the prognostic impact of *RHOA* in GC is currently unknown [67]. *CLDN18*-*ARHGAP* fusions were found in 15% of GS subtype, and were mutually exclusive from *RHOA* mutations [64].

Clustering mutations based on pathways in the GS subtype reveals interesting findings. It was discovered that there is elevated expression of mitotic network components such as AURKA/B and E2F, targets of MYC activation, FOXM1 and PLK1 signaling and DNA damage response pathways across all subtypes, but to a lesser degree in GS tumors [4]. However, the GS subtype exhibited elevated expression of cell adhesion pathways, including the B1/B3 integrins, syndecan-1 mediated signaling, and angiogenesis-related pathways [4]. These unique patterns of mutations in the GS subtype offer new candidate therapeutic targets, which warrant further investigation.

### Chromosomal instability

Chromosomal instability GCs are classified based on degree of aneuploidy, comprising approximately 50% of GC [4]. CIN is characterized by highly variable chromosomal copy numbers, without exhibiting high mutation rates. CIN subtype tumors are frequent at the gastroesophageal junction/cardia, correlate with the Lauren intestinal histologic variant, show marked aneuploidy, and harbor focal amplifications of RTKs, in addition to recurrent *TP53* mutations and RTK–RAS activation [4]. Molecular characterization has identified CIN gastric subtype to be similar to esophageal adenocarcinoma, comprising one large subgroup [68]. As the prognosis of CIN is similar to that of MSI subtype, it is worthwhile to explore targeted treatments in this subtype based on its unique molecular profile [45].

As *TP53* mutations cause chromosomal instability, it is consistent with the finding from TCGA that CIN GCs have an enrichment of *TP53* mutations and recurrent chromosomal amplifications and deletions. RTKs amplification is a signature of CIN GC. Frequent amplifications have been found in the genomic regions of RTK–RAS, which harbors *EGFR*, *ERBB2*, *ERBB3*, *MET*, *VEGFA*, and *KRAS* [4]. Hence, it is worthwhile to explore the benefits of the HER2 monoclonal antibody, trastuzumab, in CIN tumors harboring *ERBB2* amplification. Furthermore, VEGF-A inhibitors could also be used in this subgroup, as recurrent amplification of *VEGFA* was notable in the TCGA cohort. Other amplified genes in CIN include oncogenic transcription factors, such as MYC, GATA4, and GATA6, and cell cycle regulators including CCNE1, CCND1, and CDK6 [4]. Hence, cyclin-dependent kinase inhibitors could also be promising in CIN. Chromosomal deletions have also been found in CIN, in genomic regions containing *FHIT* (*Fragile histidine triad*), *WWOX* (*WW domain containing oxidoreductase*), *STK3* (*Serine/threonine kinase 3*), *CDH1*, *CTNNA1* (*Catenin alpha 1*), *PARD3* (*Par*-*3 family cell polarity regulator*), and *RB1* (*retinoblastoma 1*) [64].

Amplification of *fibroblast growth factor receptor 2* (*FGFR2*) is also frequent in CIN GCs [4], and is of considerable interest due to clinical trials investigating FGFR inhibitors. FGFR is a tyrosine kinase receptor, which binds to FGF and triggers cell growth, proliferation, differentiation, migration and survival [69]. *FGFR* amplification in GC is associated with poor prognosis and lymphatic invasion [70]. In EGJ adenocarcinoma, however, FGFR2 expression, but not amplification, is associated with poor survival [71]. The FGFR pathway has been of interest to researchers, leading to several FGFR inhibitors currently under investigation in preclinical and clinical trials, with tolerable safety profiles to date. FGFR inhibitors have been shown to enhance tumor sensitivity to conventional chemotherapeutic drugs such as 5-fluorouracil, irinotecan, paclitaxel, and etoposide [72]. Recent pharmaceutical development has led to highly selective FGFR inhibitors, including drugs such as AZD4547, which, despite encouraging preliminary results, unfortunately failed to improve progression free survival (PFS) versus chemotherapy as second-line treatment in GC with *FGFR2* amplification/polysomy [73]. Of note, the authors highlighted a considerable intra-tumor heterogeneity for *FGFR2* amplification and poor concordance between *FGFR2* amplification/polysomy and FGFR2 expression, suggesting the need for alternative biomarker testing. Another phase II study (NCT02699606) examining selective FGFR inhibitor, erdafitinib, is also ongoing with preliminary results still pending. In addition to highly selective FGFR inhibitors, clinical trials using multi-kinase inhibitors with pan-FGFR inhibition are ongoing [72]. Among these, dovitinib (TKI258) is currently being investigated in several phase I and II clinical trials (NCT01791387, NCT01719549, NCT02268435) including patients with *FGFR2* amplification and GC [69]. Overall, *FGFR2* amplification in gastroesophageal cancers presents an exciting opportunity for testing these novel drugs, thereby, improving patient prognosis and future outlooks for these patients.

## ACRG molecular subtypes

On May, 2015, the ACRG published a molecular classification of GC [5], which is based on a large sample size (300 cases) and integrated molecular data from whole-genome sequencing, gene expression profiling, genome-wide copy number microarrays and targeted gene sequencing. By the integration of the data analysis, ACRG classified GC into four distinct molecular subtypes, which are associated with distinct genomic alterations, survival outcome and recurrence patterns after surgery [74]. Importantly, they confirmed the presence of the proposed molecular subtypes in previously published GC cohorts: the TCGA gastric cohort [75] and the gastric cancer Project’08 Singapore cohort [76], which suggested that the ACRG molecular subtypes could be reproduced in other GC cohorts.

Asian Cancer Research Group gene expression signatures defined four molecular subtypes of GC, which were different from the TCGA subtypes: MSI (N = 68), epithelial-to-mesenchymal transition (microsatellite stable (MSS)/EMT, N = 46), MSS/TP53 positive (N = 79) and MSS/TP53 negative (N = 107). MSI tumors typically have an intestinal-type by Lauren classification (> 60% of subjects) and show MLH1 loss of RNA expression and an elevated DNA methylation signature, occurred predominantly in the antrum (75%), and > 50% of subjects were diagnosed at an early stage (I/II); MSS/EMT tumors typically have a diffuse-type by Lauren classification at stage III/IV, include a large set of signet ring cell carcinomas, and show CDH1 loss of expression, occurred at a significantly younger age; EBV infection occurred more frequently in the MSS/TP53+ group. In addition, the authors observed that the MSI subtype had the best prognosis, followed by MSS/TP53+ and MSS/TP53−, with the MSS/EMT subtype showing the worst prognosis of the four (log-rank, *P *= 0.0004). The MSS/EMT group had a higher chance of recurrence compared to the MSI group (63% versus 23%). When they looked at the first site of recurrence, they observed a higher percentage of subjects with peritoneal seeding in the MSS/EMT GC subtype and liver-limited metastasis in the MSI and MSS/TP53− subtypes, which may have a huge impact on the clinical practice.

Regarding the somatic mutations associated with each ACRG group, the authors observed that the MSI subtype, similar to TCGA, was associated with the presence of hypermutation, with mutations in *ARID1A* (44.2%), the *PI3K*-*PTEN*-*mTOR* pathway (42%), *KRAS* (23.3%) and *ALK* (16.3%). The EMT subtype had a lower number of mutation events when compared to the other MSS groups. The MSS/TP53− subtype showed the highest prevalence of *TP53* mutations (60%), with a low frequency of other mutations, as well as focal amplification of *ERBB2*, *EGFR*, *CCNE1*, *CCND1* whereas the MSS/TP53+ subtype showed a relatively higher prevalence (compared to MSS/TP53−) of mutations in *APC, ARID1A*, *KRAS*, *PIK3CA* and *SMAD4.* Of note, *ERBB2* amplification was seen in 17.4% of MSS/TP53− tumors, compared to MSS/TP53+ (3.0%), MSI (0.0%) and MSS/EMT (0.0%, *P *= 0.0001). These findings implied that the subtype of MSS/TP53− maybe suitable for approved HER2-targeting agent, such as trastuzumab [21].

The authors compared the ACRG subtypes with the TCGA genomic subtypes. When applied to both ACRG and TCGA data sets, they observed similarities among MSI tumors in both data sets, and they showed that the TCGA GS, EBV+ and CIN subtypes were enriched in ACRG MSS/EMT, MSS/TP53+ and MSS/TP53− subtypes, respectively. Furthermore, the authors observed that the tumors classified as the TCGA CIN subtype were present across all ACRG subtypes in the TCGA data set, while tumors classified as the GS subtype in the TCGA set were present across all ACRG subtypes in the ACRG data set. Nevertheless, the ACRG researchers saw a substantially lower percentage of Lauren’s diffuse-subtype cases in the TCGA cohort (24% in TCGA versus 45% in ACRG) with the majority (57%) of Lauren’s diffuse-subtype cases present in the TCGA GS group but only 27% cases present in the ACRG MSS/EMT subtype. In addition, although *CDH1* mutations were highly prevalent in the TCGA GS subtype (37%), they were infrequent in the ACRG MSS/EMT subtype (2.8%), suggesting that the TCGA GS type is not equivalent to the ACRG MSS/EMT subtype. Such findings suggest that the TCGA and ACRG classification systems are related but distinct [77].

## Biomarkers in gastro-esophageal cancer: where are we going

Main promising biomarkers and future directions in the field discussed in the following sections are summarized in Table 2.

**Table 2:** Promising future biomarkers

Biomarker	Description	Potential value	Refs.
HER2 loss	Loss of HER2 overexpression after anti-HER2 treatment	Predictive: secondary resistance to anti-HER2 agents	[102]
*EGFR*, *MET*, *KRAS*, *ERBB3*, *CCNE1*, *CDK6*, *CCND1, FGFR2* and *PIK3CA* alterations, loss of *PTEN*	Secondary driver alterations (mutations/amplification) co-occurrent with *HER2* amplification in GEA	Predictive: primary resistance to anti-HER2 Potential target for combined blockade	[98–101]
*MYC*, *EGFR*, *FGFR2* and *MET* amplifications	Acquired alterations under anti-HER2 treatment pressure	Predictive: secondary resistance to anti-HER2 agents Potential target for novel treatment strategies	[103, 104]
Liquid biopsy	Mutational analysis of circulating tumor DNA	Molecular profiling and identification of predictive mutations for targeted treatments at baseline Dynamic monitoring of treatment response/disease progression Early detection of secondary resistance	[111–117]
DNA methylation	Aberrant promoter DNA methylation in target genes	Diagnostic value Negative prognostic value Possible predictive value and role as novel treatment target	[118–120]
miRNA	Micro RNA: short noncoding single-stranded RNA molecules, with post-transcriptional regulatory functions	Diagnostic and prognostic value Possible predictive value and role as novel treatment target	[124–129]
lncRNA	Long noncoding RNA: noncoding single-stranded RNA molecules, > 200 nucleotides, involved in cancer development and metastases	Possible diagnostic and prognostic value Possible predictive value and role as novel treatment target	[125, 130–132]
PDX models	Patient-derived xenograft animal models with defined molecular signatures	Predictive: preclinical studies with targeted drugs	[133, 134]

### Microsatellite instability and PD-L1 status: immunotherapy in GEA

Over the last year the groundbreaking success of immunotherapy with checkpoint inhibitors has opened a new era in the treatment of MSI-H tumors, including GEA.

Based on the positive results of the KEYNOTE-059 trial, the anti-PD-1 monoclonal antibody pembrolizumab (Keytruda^®^, Merck & Co., Inc.) has been approved by the Food and Drug Administration (FDA) for the treatment of patients with programmed cell death-ligand 1 (PD-L1)-positive (> 1%) advanced GC or GEJ adenocarcinoma [78, 79]. A pre-planned analysis of the study, in fact, showed a significantly higher response rate in PD-L1-positive tumors when compared to PD-L1 negative ones.

More recently the Japanese Ministry of Health, Labor and Welfare (MHLW) approved nivolumab (Opdivo^®^, Bristol-Myers Squibb), another anti-PD-1 monoclonal IgG4 antibody, for the treatment of unresectable advanced or recurrent GC progressing after chemotherapy. The approval was based on positive results of the phase III ATTRACTION-2 (ONO-4538-12) trial, enrolling 493 Asian patients with advanced or recurrent gastric or GEJ cancer refractory to at least two previous chemotherapy. This study showed a significant reduction in patients’ risk of death and an increased overall survival (OS) rate at 12 months from nivolumab when compared to placebo [80]. Notably, no predictive biomarker has been required for this indication.

Data on immunotherapy in esophageal cancer are available as well. In the multicohort phase Ib KEYNOTE-028 trial, in fact, pembrolizumab as single agent has been tested also in a series of PD-L1-positive esophageal cancer after failure of standard chemotherapy (including both squamous cell carcinomas and EAC). Promising results showed an overall response rate of 30.4 and 52.2% in squamous cell carcinoma and EAC, respectively, with a 12-month progression free rate of 21.7%, in an heavily pretreated patient population [81].

On the other hand, anti-CTLA-4 agents monotherapy (i.e. ipilimumab and tremelimumab) showed higher toxicity and lower efficacy than anti-PD-1 in EGA [82, 83]. Combined therapy with anti-CTLA-4 antibodies and anti-PD-1, however, have been tested with encouraging results and is currently the object of further investigation (NCT02872116).

Additionally, pembrolizumab is currently under investigation in several different settings. The phase III KEYNOTE-06 compared pembrolizumab versus paclitaxel as second-line treatment in patients with advanced GC [84]; recently released updates from this study showed no significant benefit in this setting. The ongoing phase III KEYNOTE-062 is testing pembrolizumab as a monotherapy and in combination with chemotherapy in the first-line treatment of PD-L1–positive advanced GC or GEJ cancer, while the phase III KEYNOTE-585 is evaluating the combination of pembrolizumab with chemotherapy in the neoadjuvant and adjuvant settings.

Another strategy under study for immunotherapy in GEA is targeting PD-L1. Avelumab, a fully human anti-PD-L1 IgG1 antibody, has been investigated as a first-line maintenance or second-line treatment in patients with advanced GC or GEJ cancer with promising results (NCT01772004) [85]. Unfortunately, it was recently announced that the phase III JAVELIN Gastric 300 trial (NCT02625623), investigating avelumab as a third-line treatment advanced GC and GEJ adenocarcinoma, unselected for PD-L1 expression, failed to meet its primary endpoint (OS) [86]. Results of the phase III JAVELIN Gastric 100 trial evaluating avelumab as first-line maintenance therapy following induction chemotherapy in gastric or GEJ cancer are awaited.

The relevance of testing for MSI-H/dMMR status or PD-L1 expression, thus, has become crucial in GEA since eligible patients can now receive immunotherapy as a standard treatment. Of note, based on recent molecular subtypes, EBV-positive and MSI-H GCs emerge as the best candidates for immunotherapy based on the increased PD-L1 expression associated with these subtypes and the high tumor mutational load in MSI-H GEA, which has been shown to correlate with a greater benefit from anti-PD-1/PD-L1 blockade [87]. Nevertheless, new strategies and novel therapeutic targets are needed to increase treatment options for GEA patients. A promising novel biomarker, V-domain Ig suppressor of T-cell activation (VISTA), also known as PD1 homolog (PD1H), has been recently analyzed in GC. VISTA expression was present in 8.8% out of 464 analyzed samples, and was associated with clinical and molecular features such as Lauren phenotype, tumor localization, EBV infection, *KRAS* and *PIK3CA* mutational status and PD-L1 expression [88]. A combined blockade of VISTA and PD-1 might thus be a promising treatment option in these patients,

Several additional clinical trials investigating the efficacy of immune checkpoint inhibitors in GEA, in different settings and exploring different treatment strategies and combinations with other agents, are ongoing and can be found reviewed in dedicated papers [89–93].

### Beyond HER2: primary and acquired resistance to trastuzumab

As aforementioned, trastuzumab in combination with chemotherapy has been the first targeted therapeutic to demonstrate a survival improvement in patients with advanced HER2-positive GEA. However, not all HER2-positive patients respond to trastuzumab and most patients eventually develop an acquired resistance to this drug during treatment. Furthermore, alternative targeted anti-HER2 strategies, conversely from other tumor types (i.e. HER2-positive breast cancer) failed to show significant benefit in the treatment of GEA. For instance, both lapatinib, an oral tyrosine kinase inhibitor (TKI) dual inhibitor of HER2 and EGFR, and trastuzumab-emtansine (T-DM1), an antibody-drug conjugate of trastuzumab and emtansine a microtubule inhibitor, failed to show a significant survival improvement in addition to chemotherapy compared to chemotherapy alone in this setting [94–96]. Other agents such as pertuzumab and afitinib are currently being evaluated in a phase III (NCT01358877, NCT01774786) and a phase II (NCT01522768) clinical trial, respectively, in the second-line setting following first-line trastuzumab therapy, after the addition of pertuzumab to trastuzumab plus chemotherapy in the first-line setting failed to demonstrate a significant survival benefit [97]. Although the exact mechanisms underlying primary and acquired resistance to HER2 targeted therapy are still under study, intra-tumor heterogeneity and the activations of downstream signaling pathways including several RTKs seem to be involved in tumor escape from HER2-blockade.

As highlighted in previous sections, recent genomic studies have disclosed the high degree of complexity of GEA genomic landscape underlining the challenges of biomarker assessment in these tumors. Several secondary alterations in key cancer genes have been reported to occur frequently in HER2-positive GEA. Among these *EGFR*, *MET*, *ERBB3*, *CCNE1*, *CDK6*, *CCND1*, and *PIK3CA* [98]. Notably the co-occurrence of these alterations has been showed to confer resistance to HER2-targeted treatment in vitro, which can be reversed by combined blockade of HER2 and secondary driver mutations, thus suggesting a promising rationale for combined targeted therapies to overcome primary HER2 resistance in HER2 positive tumors. Indeed, in a small case series, a patient with a co-amplification of HER2 and MET was treated with a combination of trastuzumab, crizotinib, and paclitaxel and experienced near-complete disease response [99], and combined targeted blockade warrants further investigations. Additionally, loss of PTEN expression and low HER2 amplification index have been correlated with primary resistance to first-line trastuzumab-based therapy and poor prognosis in a study involving 129 HER2 positive GC [100].

More recently Pietrantonio et al. reported the results of a study investigating biomarkers of primary resistance to trastuzumab in HER2-positive metastatic GC (the AMNESIA study). A panel of candidate genomic alterations including *EGFR*, *MET*, *KRAS*, *PI3K* and *PTEN* mutations and *EGFR*, *MET*, and *KRAS* amplifications was tested in 37 patients treated with trastuzumab (17 responders and 20 patients with primary resistance). AMESIA panel alterations were significantly more frequent in resistant patients and in HER2 IHC 2+ compared to HER2 IHC 3+ tumors. The absence of any alteration was correlated with longer median PFS and OS and the predictive accuracy of the combined evaluation of the AMNESIA panel and HER2 IHC was 84% [101]. These promising results, however, need further prospective validation.

In another previous study, the same author explored the possible mechanisms of anti-HER2 acquired resistance in GEA. In a small series of 22 matched pre-treatment and post-progression samples from patients receiving chemotherapy and trastuzumab for advanced HER2‐positive (IHC 3+ or 2+ with ISH amplification) GEA, HER2 loss was identified a mechanism of resistance in 32% of cases. Notably, the chance of HER2 loss was not associated with any baseline clinico-pathological features except initial IHC score 2+ versus 3+ [102]. Loss of HER2 overexpression might partially explain the failure of second-line anti-HER2 treatment strategies in initially HER2-positive tumors.

Additionally, molecular alterations emerging upon tumor progression after trastuzumab treatment have been observed in several candidate genes such as *TP53* (92%), *EGFR* (13%), cell-cycle mediators, i.e. cyclin-dependent kinases (42%) and in the PI3K/AKT/mTOR axis (21%) [103]. Similarly, a recently reported biomarker analysis from a phase II study evaluating the efficacy of lapatinib in combination with chemotherapy as first-line treatment in HER-2-positive GC showed the emergence of genomic aberrations such as *MYC*, *EGFR*, *FGFR2* and *MET* amplifications at disease progression [104].

None of these biomarkers, however, are currently implemented in clinical practice, and additional evidence is necessary to optimize patient selection and personalize treatment strategies based on the definition of key mechanisms of resistance to targeted treatment and development of effective alternative targeted therapies for refractory disease, including combined targeted blockade of concomitant or emerging secondary driver alterations.

### Molecular heterogeneity between primary tumor and metastatic disease: possible role of liquid biopsy

Intra-tumor heterogeneity has been shown to be extremely relevant in GEA. Indeed, HER2 expression has been found to range widely with variable percentages of tumor cells staining positive in the same samples, and variable concordance rates between biopsy and paired surgical resections have been reported [105, 106], as well as previously discussed changes in HER2 expression related to targeted treatment (HER2 loss). Additionally, recent results of a large-scale profiling study in GC confirmed a high grade of tumor heterogeneity in EBV-positivity and *PIK3CA* mutations, suggesting caution in the extrapolation of tumor genomic profiling from the analyses of single tissue biopsies [107]. Discordance in HER2 expression between primary tumor (PT) and metastatic lesions (MLs) has been reported as well [108, 109], possibly due to a clonal selection during tumor progression or to intra-tumor heterogeneity of HER2. These data underline the issue of possible limitations in molecular testing in GEA due to single specimen analyses which might not be representative of the whole tumor genetic landscape.

More recently two works highlighted a deeper level of genomic heterogeneity between PTs and MLs in GEA through the use of targeted NGS and whole-exon sequencing techniques [110, 111]. Of note, Pectasides and colleagues sequenced paired primary GEA and MLs across multiple cohorts, finding remarkable levels of discordance in genomic alterations, including potentially clinically relevant alterations, reaching up to 60% for the amplification profile of genes such as *HER2*, *EGFR*, *KRAS* and *CDK4/6*. Their study included a pilot analysis of cell-free DNA (cfDNA) which showed both concordance and discordance with matched PT and MLs results, as sequencing of cfDNA was able to identify in some cases alterations (i.e. genomic amplifications) not observed in the PTs, but at the same time failed in other cases to show the presence of known alterations involving genes such as HER2 and FGFR found in PTs. Additionally, profiling of paired PTs, MLs, and cfDNA from patients enrolled in the PANGEA (Personalized Antibodies for Gastroesophageal Adenocarcinoma) trial (NCT02213289) highlighted a recurrent discrepancy of genomic biomarkers between PTs and untreated metastases, which led to treatment reassignment in about one-third of patients. In case of discordant PT and MLs, cfDNA displayed an 87.5% concordance rate with MLs for targetable alterations, suggesting the potential role of cfDNA testing to enhance targeted treatment selection [111].

Indeed, several studies have underlined the promising role of ctDNA testing (commonly referred to as ‘liquid biopsy’) as a less invasive and more comprehensive method to pharmacogenomic profiling and dynamic molecular monitoring in several cancer types, including GEA. Notably, a study from Gao et al. demonstrated that the mutational profile of ctDNA in a series of 30 GC patients was able to reflect the sum of somatic mutations present in multiple paired tissue samples while the concordance with a single tumor sample was low, highlighting once again the issue of tumor heterogeneity in GEA and the potential of ctDNA to at least partially overcome it [112]. Additionally, in this study *HER2* amplification in ctDNA were showed to be highly concordant with *HER2* amplification in tumor tissue. Furthermore, Wang and colleagues recently reported positive data on the use of ctDNA to evaluate HER2 copy number levels as a minimally-invasive biomarker to predict and monitor trastuzumab efficacy in advanced GC [113]. Main evidence on liquid biopsy in GC are reviewed in dedicated papers [114–116]. Of note, recent evidence suggests a prognostic value as well as a role in monitoring treatment response and risk of recurrence, for ctDNA in early stage esophageal cancers [117].

Although validation and further investigations are critical, altogether these data support the role of liquid biopsy as a promising technique for genomic profiling, targeted treatment selection and monitoring of treatment response as well as early detection of secondary resistance mechanisms in GEA, which warrants further development for future clinical applications.

### Emerging role of epigenomics and miRNA in GEA

Epigenetic changes, including DNA methylation, histone modifications and non-coding RNAs, are a common event in cancer and contribute to both carcinogenesis and disease progression. Aberrant DNA methylation is one of the most studied epigenetic alteration in cancer and it has been proposed as a potential biomarker both for tumor diagnosis, prognosis and treatment response in several cancer types.

Promoter DNA methylation of several tumor suppressor genes has been reported in pre-malignant stages of GC, suggesting a potential role for early cancer detection of these biomarkers, which have been identified either in blood, gastric juice or stool samples (reviewed in [118]). On the other hand, promoter hypermethylation of several genes has been associated with worse prognosis in GC (reviewed in [119]). Notably, both *Helicobacter pylori* and EBV infections are associated with increased levels of DNA methylation, and, as previously discussed, EBV-positive tumors exhibit extreme CIMP signature involving hypermethylation of numerous target genes. In EAC, abnormal DNA methylation has been extensively researched as a tool for stratifying Barrett’s esophagus patients’ risk to develop cancer. Aberrant methylation in several genes, in fact, such as *CDKN2A* and *APC*, has been reported as part of the neoplastic progression from Barret’s esophagus to EAC [120]). Similar with GC, promoter methylation of multiple genes has been associated with poor prognosis in EAC. Future research will further address the promising diagnostic and prognostic value of aberrant DNA methylation in GEA and its possible implication in treatment response as well as its potential role as a treatment target in these malignancies.

In recent years, miRNAs have emerged as critical regulators in the oncogenesis pathways and have been proposed as useful novel diagnostic and prognostic biomarkers in multiple cancer types [121, 122]. These small noncoding RNA fragments regulate target genes expression by binding to their 3′UTR region and impairing translation, hence modulating a broad range of biological processes comprising cellular signaling, metabolism, apoptosis, proliferation and differentiation, acting either as oncogenes or as tumor suppressors [123]. Their role as a biomarker represents an expanding field of research in GEA [124–127]. Several miRNAs have been identified and implicated in GC and EAC diagnosis and prognosis and many others are currently under investigation [128, 129]. A predictive role of miRNAs in treatment response has been proposed as well. Although available data still need validation, the possible clinical application of miRNAs as biomarkers or as a potential target of treatment in GEA deserves further investigation.

In addition to miRNA, long noncoding RNAs (lncRNAs), are recently becoming one of the next frontier of cancer research. Recent findings, in fact, suggest that they play an important role in carcinogenesis and metastasis and numerous lncRNAs have been found to be altered in GEA, thus supporting a strong rational for their potential role as biomarkers in these malignancies [125, 130–132].

### Patient-derived xenograft models

Patient-derived xenograft (PDX) models represent a novel approach with the potential to enhance biomarker discovery and preclinical testing of personalized treatment options, providing a platform which replicates tumor molecular and biological features as well as tumor microenvironment in the animal model.

Patient-derived xenograft models have been successfully created for GC and explored in several studies. Notably, PDX models of tumors harboring alterations in HER-2, MET and FGFR2 signaling pathways have been proven useful for targeted drugs screening and evaluation, highlighting preliminary evidence of activity of the combination of targeted anti-MET and anti-FGFR2 treatment in tumors with co-occurrent amplifications of these genes [133]. More recently, tumor molecular profiling of PDX models has been used to guide treatment selection and test the efficacy of selected targeted drugs while exploring possible candidate response biomarkers [134]. The authors of this study were able to identify definite molecular signature in different PDX models with corresponding individual histopathological and molecular features. Main recurrent genomic alterations involved the MAPK, ErbB, VEGF, mTOR, and cell cycle signaling pathways. Several potential drug targets were selected and activity of targeted blockade (i.e. anti-MET volitinib, anti-EGFR monoclonal antibody BK011 and cetuximab, afatinib, apatinib and the CDK1/2/9 inhibitor AZD5438), was demonstrated in corresponding models. These preliminary results should be validated in larger studies with PDX models or in clinical trials, nevertheless current evidence supports future perspectives on a wider use of PDX models with defined molecular signatures in preclinical studies with targeted drugs.

## Conclusions

As our knowledge on the genomic landscape of GEA continues to evolve, uncovering the high heterogeneity and deep complexity of these tumors, current efforts are centered upon establishing the clinical relevance of novel molecular subtypes and validate novel biomarker-driven targeted treatment approaches.

The availability of new technologies and the identification of promising novel biomarker with the potential to overcome tumor heterogeneity and provide a dynamic monitoring of tumor molecular evolution under treatment pressure will be crucial to optimize drug development and clinical investigation in a setting where therapeutic options are currently lacking.

Several promising biomarkers are under study and growing evidence is accumulating on mechanisms of primary and acquired resistance to treatment, nevertheless further validation is necessary before translating available evidence into clinical practice.

## Abbreviations

ACRG: Asian Cancer Research Group
AKT: AKT8 virus oncogene cellular homolog
Ang-2: angiopoietin-2
B2M: beta-2-microglobulin
BMP: bone morphogenetic protein
BRAF: v-Raf murine sarcoma viral oncogene homolog B1
CDH1: cadherin 1
CIN: chromosomal instability
CIMP: CpG island methylation phenotype
cfDNA: circulating free DNA
ctDNA: circulating tumor DNA
CTLA-4: cytotoxic T lymphocyte-associated protein 4
CTNNA1: catenin alpha 1
EAC: esophageal adenocarcinoma
EBV: Epstein-Barr virus
EC: esophagus carcinoma
EGFR: epidermal growth factor receptor
ERK: extracellular signal-regulated kinase
FDA: Food and Drug Administration
FGFR2: fibroblast growth factor receptor 2
FHIT: fragile histidine triad
FISH: fluorescent in situ hybridization
GEA: gastro-esophageal adenocarcinomas
GEJ: gastro-esophageal junction
GC: gastric cancer
GS: genomically stable
HER2/neu: human epidermal growth factor receptor 2
IHC: immunohistochemical staining
KRAS: Kirsten rat sarcoma viral oncogene homolog
lncRNA: long noncoding RNA
MAPK: mitogen-activated protein kinase
MEK: mitogen-activated protein kinase kinase
MET: tyrosine-protein kinase Met
MHLW: Japanese Ministry of Health, Labor and Welfare
miRNA: micro RNA
MMR: mismatch repair
MSI: microsatellite instability
MSS: microsatellite stable
mTOR: mammalian target of rapamycin
NGS: next generation sequencing
OS: overall survival
PARD3: Par-3 family cell polarity regulator
PD-1: programmed cell death protein 1
PD1H: PD1 homolog
PD-L1: programmed death-ligand 1
PDX: patient-derived xenograft
PFS: progression free survival
PIK3CA: phosphatidylinositol 3-kinase catalytic subunit alpha
PTEN: phosphatase and tensin homolog
RAF: v-Raf murine sarcoma viral oncogene homolog
RAS: rat sarcoma viral oncogene homolog
RB1: retinoblastoma 1
RET: REarranged during transfection
ROHA: Ras homolog family member A
RTK: receptor tyrosine kinase
STK3: serine/threonine kinase 3
TCGA: The Cancer Genome Atlas
T-DM1: trastuzumab-emtansine
TILs: tumor infiltrating lymphocytes
TKI: tyrosine kinase inhibitor
VEGF: vascular endothelial growth factor
VEGFR: VEGF receptor
VISTA: V-domain Ig suppressor of T cell activation
WWOX: WW domain containing oxidoreductase)
